# Leptin-induced inflammation by activating IL-6 expression contributes to the fibrosis and hypertrophy of ligamentum flavum in lumbar spinal canal stenosis

**DOI:** 10.1042/BSR20171214

**Published:** 2018-03-29

**Authors:** Chao Sun, Zhen Wang, Ji-Wei Tian, Yun-Hao Wang

**Affiliations:** 1 Department of Spine Surgery, Shanghai General Hospital of Nanjing Medical University and the Affiliated Jiangning Hospital of Nanjing Medical University, Nanjing 211100, China; 2 Department of Orthopedics, Shanghai General Hospital of Nanjing Medical University, Songjiang 201600, Shanghai, China

**Keywords:** Fibrosis, Hypertrophy, Ligamentum flavum, Lumbar spinal canal stenosis, Leptin

## Abstract

The ongoing chronic inflammation and subsequent fibrosis play an important role in ligamentum flavum (LF) fibrosis and hypertrophy in patients with lumbar spinal canal stenosis (LSCS). Leptin is a chronic inflammatory mediator and involved in the fibrotic process in multiple organ systems. The present study aimed to investigate the role of leptin in LF fibrosis and its related regulatory mechanisms. The LF specimens were obtained during the surgery from 12 patients with LSCS (LSCS group) and 12 control patients with lumbar disc herniation (LDH) group. The morphologic changes and fibrosis score of LF were assessed by Hematoxylin and eosin (H&E) and Masson’s trichrome staining respectively. The location and expression of leptin in LF tissues were determined. Then, the LF cells were cultured and exposed to recombinant human leptin (rhleptin). Collagen I and III were used as fibrosis markers and IL-6 was used as the inflammatory factor. As a result, the LF thickness and fibrosis score in the LSCS group were significantly higher than those in the LDH group (*P*<0.05). Leptin was detected in the hypertrophied LF and its expression was substantially increased in the LSCS group and positively correlated with LF thickness and fibrosis score (*P*<0.05). Moreover, our *in vitro* experiments revealed that rhleptin treated LF cells elevated the expression of collagen I and III. Finally, leptin administration induced IL-6 expression via nuclear factor-κB (NF-κB) pathway in LF cell (*P*<0.05). Our study demonstrated novel molecular events for leptin-induced inflammation in LF tissue by promoting IL-6 expression and thus might have potential implications for clarifying the mechanism underlying LF fibrosis and hypertrophy.

## Introduction

Lumbar spinal canal stenosis (LSCS) is one of the most common diseases in the elderly population often with symptoms of low back and leg pain, numbness, and intermittent claudication arising from nerve compression [1]. Numerous causative factors such as disc protrusion, the bony proliferation of the facet joints, and hypertrophy of the ligamentum flavum (LF) contribute to the development of LSCS. It is important to note that LF hypertrophy is viewed as the important cause of LSCS [2].

In the normal LF, elastic fibers consists of 80% in the extracellular matrix [3]. However, the hypertrophied LF is characterized by the loss of elastic fibers and an increase of collagen fibers, exhibiting fibrotic changes [4,5]. Importantly, fibrosis is reported to be the main cause of LF hypertrophy [3,5]. Previously, several studies have explored the mechanism of LF fibrosis at the histological and cellular levels, and some genetic and biological factors have been ascertained. Nonetheless, the exact molecular mechanism remains poorly understood.

Leptin, namely the obese gene, is a 16 kDa peptide hormone product of cytokine family. Since the first identification in 1994 by Zhang et al. [6], the role of leptin has been widely reported by previous studies [7–11]. Its main function is a central mediator which is associated with the development of obesity and metabolic syndrome [7]. Recently, it has been demonstrated that multiple tissues and cells can produce and secrete leptin, suggesting other important biological functions beyond appetite regulation and energy metabolism [8]. Interestingly, accumulating evidence suggests that it has a critical role in the fibrosis process in multiple organ systems, including the liver, kidney, and lung [9–11]. However, there are no previous studies investigating the correlation of leptin in the development of fibrosis and LF hypertrophy. Therefore, the aim of the present study was to measure the expression of leptin in human LF tissues from patients with LSCS and to explore the role of leptin in LF fibrosis and its possible regulatory mechanisms.

## Materials and methods

### Study population

A prospective study was performed on patients underwent surgery owing to LSCS in the Shanghai General Hospital from April 2017 to July 2017, which was approved by the Ethics Committee of Nanjing Medical University. For the study group, patients with degenerative LSCS underwent posterior decompressive laminectomy were selected. Diagnostic criteria of LSCS were determined based on both clinical symptoms by asking the detailed clinical history and radiological examinations. The inclusion criteria were as follows: age between 50 and 75 years; the level of L4/5 stenosis; degenerative LSCS with LF hypertrophy. Patients with other lumbar spine diseases such as scoliosis, history of congenital deformities, skeletal dysplasia, and spinal tuberculosis were excluded from the study. LF specimens were obtained during the surgery. As controls, LF specimens used in the study were harvested from age- and gender-matched patients with lumbar disc herniation (LDH) operatively managed for this disorder. Patients in this group were confirmed without LF hypertrophy using MRI. Before the surgery was undertaken, three specialists in spinal surgery assessed and advised as to whether the LF specimens were suitable for inclusion in the study. All patients provided written informed consent prior to participate in the present study.

### Measurement of LF thickness

The LF thickness was measured at the facet joint level on the T1-weighted MRI through the PACS system (a picture analyzing system, China) [12]. The maximum value was measured three times and the average value was viewed as the LF thickness.

### Histologic analysis

**Figure 1:**
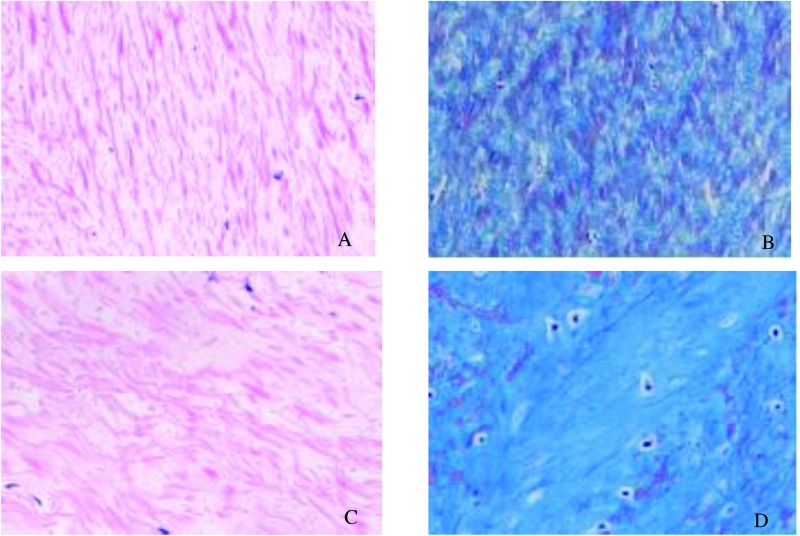
Histological analysis of LF specimens (**A**) In LF from the LSCS group, a large area was stained pink with a regular arrangement (H&E staining, ×200). However, in the LDH group (**C**), elastic fibers were disorganized and focally lost. Grading of LF fibrosis by Masson’s trichrome staining (**B** and **D**). The blue color indicates collagen fibers and the pink color indicates elastic fibers. (**B**) Grade 1 showed a large area was stained pink (×200). (**D**) In Grade 4, blue stained most of the area, indicating fibrotic change (×200).

All collected LF samples used for the subsequent experiments were only taken from the dorsal layer of LF. Hematoxylin and eosin (H&E) staining was used to observe the morphologic changes such as structure of the LF and the degree of elastin degradation. Masson’s trichrome staining was done to evaluate the degree of fibrosis. Briefly, the LF specimens obtained during the surgery were immediately fixed in 10% formalin for 48 h and embedded into the paraffin. Then, paraffin sections with thickness of 4 μm were prepared and stained with H&E staining and Masson’s trichrome staining. As previously stated, the criterion of LF fibrosis score was as follows: Grade 0 indicated normal tissue showing collagen in <20% of the entire area; grade 1 indicated fibrosis at ≤25% of the entire area; grade 2 represented fibrosis involving 25–50% of the area; Grade 3 indicates between 50% and 75%, and grade 4, over 75% [5]. Figure 1 displayed two typical cases with fibrosis of grades 1 and 4.

### Immunohistochemistry

The LF specimens were fixed in 10% neutral formalin and embedded in paraffin. Thick sections were cut into 5 μm thickness, dewaxed in xylene, and rehydrated in a graded series of alcohol solutions. Sections were then incubated with primary rabbit anti-leptin (Abcam, ab16227, U.S.A.), anti-collagen I (Abcam, ab34710, U.S.A.), and anti-collagen III (Abcam, ab7778, U.S.A.) monoclonal antibody at an optimum dilution recommended by the manufacturers, followed by incubation with the respective secondary antibody (Jackson, U.S.A.) at room temperature. Immunolabeling was performed using enhanced chemiluminescence reagents (Amersham Biosciences, Netherlands). Quantitative data were obtained using the Image software (Adobe PhotoShop).

### LF cell culture

The cells were obtained from the LF tissues as stated previously [13]. The LF specimens were harvested from patients during surgery and washed clearly with phosphate-buffered saline (PBS, Invitrogen, Canada). The tissue surrounded was removed carefully under the microscope. Then, they were minced into approximately 0.5 mm^3^ pieces and digested using 0.2% type I collagenase (Sigma) at 37°C for 1 h in serum-free Dulbecco’s modified Eagle’s medium (DMEM, Gibco, Australia), and subsequently washed by serum-containing DMEM aiming to remove collagenase activity. Finally, the specimens were incubated at 37°C in DMEM supplemented with 10% fetal bovine serum (Sigma, U.S.A.), 100 U/ml penicillin, and 100 pg/ml streptomycin (Sigma, U.S.A.). The culture medium was changed every 2 days. When the cells grew confluence in the dishes, they were digested and passaged using 0.25% trypsin. P2 generation cells were collected and used for the following experiments.

### Cell treatment

Cells derived from the LF specimens were harvested from the dishes using 0.25% trypsin for further passaging when they were grown to confluence in 60 mm culture dishes. P2 generation cells were used in the subsequent experiments. In order to analyze IL-6 and collagen expression regulated by leptin, P2 LF cells were cultured and stimulated with different concentrations of recombinant human leptin (Abcam, U.S.A.) ranging from 0 to 150 ng/ml for 24 h. Finally, cells and medium were then harvested for mRNA and protein analysis. IL-6 protein in supernatants of cell culture was measured using ELISA kit (Takara, China) according to the manufacturer’s instructions. For all cell inhibition experiments, 10^5^ cells were seeded in each well of 24-well plates and 10^6^ cells were seeded in each well of six-well plates. When cells were grown to 80% confluence, the indicated doses of different inhibitors (BAY11-7082 [14], Sigma, U.S.A. and Tocilizumab [15], Sigma, U.S.A.) under concentrations without cytotoxicity were used to stimulate cells. Forty eight hours later, cells were collected for RNA isolation, Western blot, and other subsequent experiments.

### Fluorescent quantitative PCR

Total RNA was extracted from LF tissues and cells using the Trizol reagent (Invitrogen) and converted to cDNA using the Reverse Transcription Synthesis Kit (Takara, Dalian, China). Then, the gene expression levels of leptin, collagen I and collagen III, IL-6, and GAPDH were analyzed by fluorescent quantitative polymerase chain reaction (PCR) using a Thermal Cycler Dice Real-Time system (Takara, Dalian, China). Values were normalized to glyceraldehyde-3-phosphate dehydrogenase (GAPDH) using the 2^−Δ*C*^_t_ method. The detailed of the primers were listed in Table 1.

**Table 1:** RT-PCR primers used in the present study

Primer	Sequence	Size (bp)	Annealing temperature (°C)
Leptin	Forward:5′-GGACTTCATTCCTGGGCTCC-3′	134	60
	Reverse:5′-GGAGGTTCTCCAGGTCGTTG-3′		
Collagen I	Forward:5′-CCAAGACGAAGACATCCCACCA-3′	108	60
	Reverse:5′-CCGTTGTCGCAGACGCAGAT-3′		
Collagen III	Forward:5′-TTCCTTCGACTTCTCTCCAGCC-3′	201	60
	Reverse:5′-CCCAGTGTGTTTCGTGCAACC-3′		
IL-6	Forward:5′-GTAGTGAGGAACAAGCCAGAGC-3′	235	60
	Reverse:5′-TACATTTGCCGAAGAGCCCT-3′		
GAPDH	Forward:5′-CACCCAGCACAATGAAGATCAAGAT-3′	317	60
	Reverse:5′-CCAGTTTTTAAATCCTGAGTCAAGC-3′		

### Western blotting

Western blotting was performed as described previously [13]. Briefly, total protein was extracted by Protein Extraction Sample Kit (Sigma, China) and the protein concentration was determined using the BCA protein assay (Beyotime, Jiangsu,China). Proteins were separated by SDS/PAGE and then converted to a nitrocellulose membrane (Sigma, U.S.A.) by electroblotting. The membranes were closed for 2 h at room temperature with 5% skim milk (Sigma, U.S.A.) to block nonspecific binding. The target proteins of rabbit polyclonal anti-Leptin (Abcam, ab16227, U.S.A.), p65 (rabbit anti-human, Abcam, U.S.A.), rabbit collagen I monoclonal antibody (Abcam, ab34710, U.S.A.), rabbit collagen III monoclonal antibody (Abcam, ab7778, U.S.A.), and mouse anti-GAPDH antibody (Bioworld, U.S.A.) were then added and allowed to incubate overnight at 4°C with gentle shaking, followed by incubation with 1:5000 goat anti-rabbit or anti-mouse secondary antibodies for 2 h at room temperature (Bioworld, U.S.A.). After careful washing, bands were detected using a western blotting chemiluminescence kit (Bioworld). GAPDH was used as the internal control.

### Statistical analysis

Data were presented as mean ± standard error of the mean. Statistical analyses were done using SPSS 17.0. Different groups were compared by using Student’s *t*-test. Pearson correlation analysis was conducted to determine the correlations among LF thickness, fibrosis score, and leptin concentration. We did analyses of multiple groups by one-way or two-way ANOVA. *P* value of < 0.05 was considered to be statistically significant.

## Results

### Demographic characteristics

Twelve patients with LSCS and twelve patients with LDH were recruited into the present study. Their basic profiles, including age, gender, and the level of the LF samples in two groups were shown in Table 2. No significant differences were observed regarding these variables between the two study groups using Student’s *t*-test (*P*>0.05).

**Table 2:** Profiles of patients

Index	LSCS group	LDH group
Number of patients	12	12
Mean age (years)	62.8 ± 3.6	57.9 ± 8.5
Gender (male/female)	7/5	6/6
Level	L4/5	L4/5

### LF fibrosis score and thickness

As shown in Figure 1, rich elastic fibers were arrayed in a regular order in the LDH group (Figure 1A). However, in the LSCS group, the elastic fibers were fragmented, uneven, disorganized, and focally lost (Figure 1C). Masson’s trichrome staining showed rich elastic fibers were observed in the LDH group, a large area of which was stained pink, indicating a normal LF (Figure 1B). But in the LSCS group, most of the area was stained blue, exhibiting fibrosis change (Figure 1D). The fibrosis score of the dorsal side of LF in the LSCS group was 3.1 ± 0.7, while that of group LDH was 2.3 ± 0.8. This difference was significant (*P*< 0.05).

**Figure 2:**
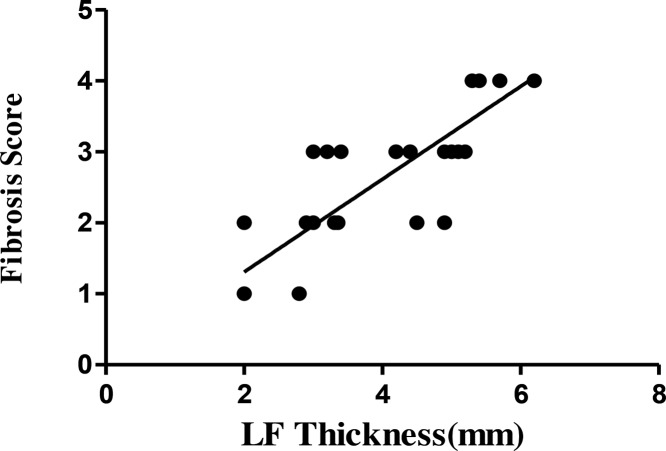
Correlation between LF thickness and fibrosis score The fibrosis score increased with LF thickness, with a significant positive correlation.

It was measured that LF thickness in the LSCS group was 5.4 ± 0.6 mm, which was significantly higher than 2.9 ± 0.6 mm in the LDH group (Table 2, *P*<0.05). Furthermore, correlation analysis showed that the fibrosis score had positive correlation with the LF thickness (*r* = 0.694, *P*<0.05) (Figure 2).

### Immunohistochemical analysis

**Figure 3:**
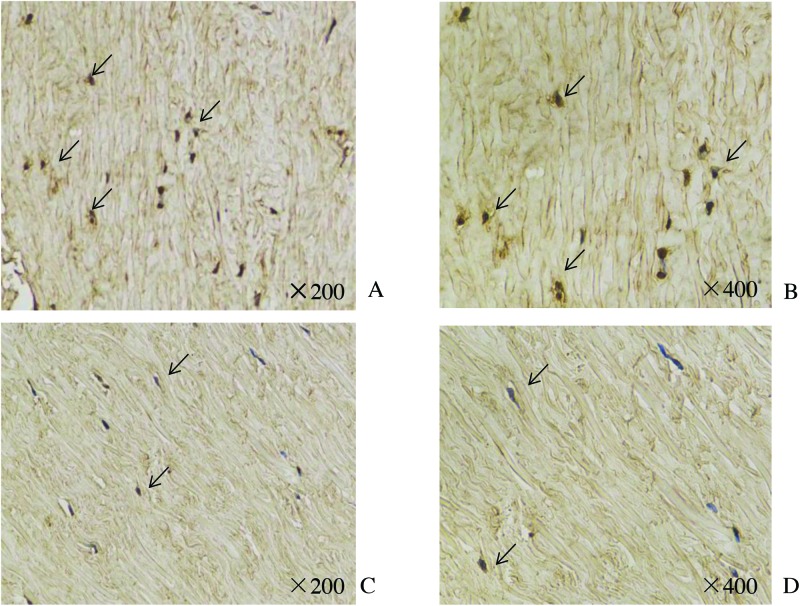
Immunohistochemical analysis of leptin in LF tissue. Results show positively stained leptin on the ligamentum flavum fibroblasts of patients with spinal stenosis. Black arrows indicate leptin-positive cells. The LSCS group (**A** and **B**) showed higher positivity than the LDH group (**C** and **D**).

We next evaluated the cellular source of leptin by immunohistochemical analysis of LF tissues. We found a markedly increased number of leptin-expressing cells in hypertrophied LF tissue from the LSCS group (Figure 3A,B) relative to normal LF tissue from the LDH group (Figure 3C,D).

### Gene expression of leptin in LF tissues

**Figure 4:**
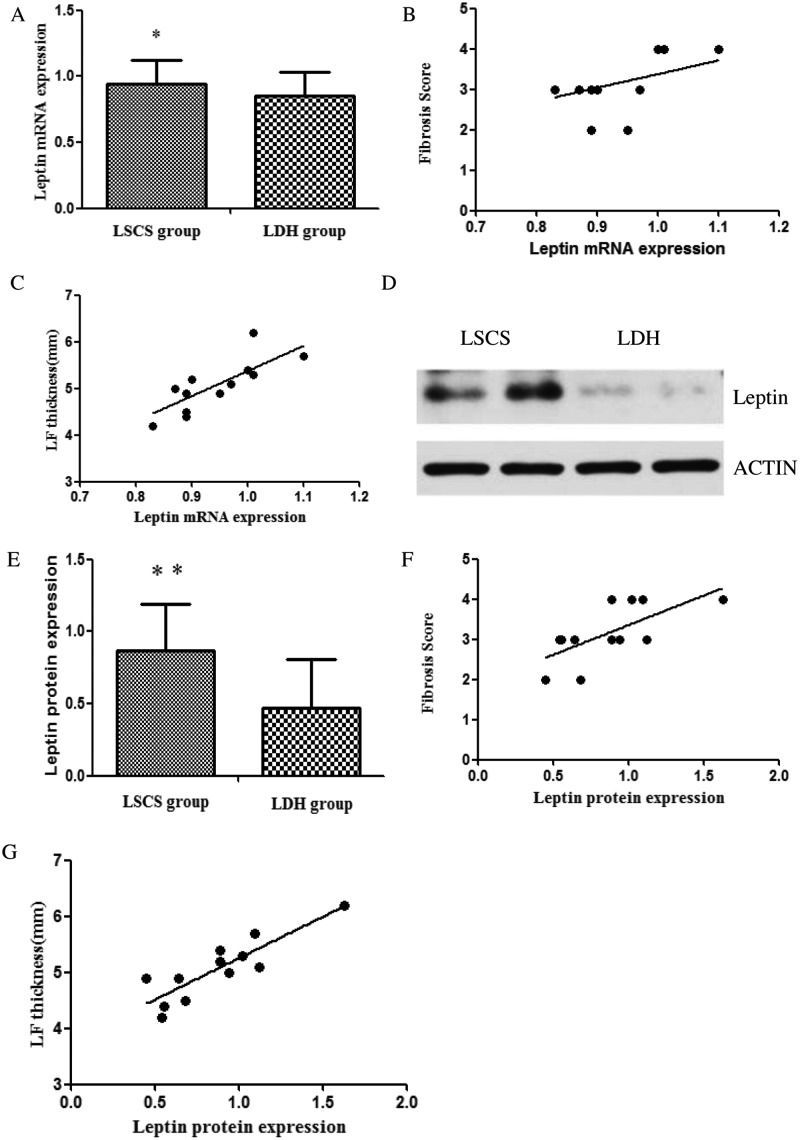
The mRNA and proten expression of leptin Both the mRNA (**A**) and protein (**D** and **E**) expression of leptin of LF tissue from patients in LSCS group was higher than that in the LDH group. Correlation analysis showed the leptin mRNA and protein expression exhibited positive correlations with the fibrosis score (**B** and **F**) and LF thickness (**C** and **G**); *P<0.05, **P<0.01.

Both the mRNA and protein expression levels of leptin were increased significantly in LF samples from LSCS group as compared with the LDH group (Table 3 and Figure 4, *P*<0.05). Furthermore, correlation analysis showed that leptin mRNA exhibited a positive correlation with the LF thickness (*r* = 0.799, *P*<0.05) and fibrosis score (*r* = 0.646, *P*<0.05); however, the slope was relatively gentle. Leptin protein expression showed a very strong correlation with LF thickness (*r* = 0.870) and fibrosis score (*r* = 0.677), and the association was shown to be statistically significant (Figure 4, *P*<0.01).

**Table 3:** Comparison of data between two groups

Index	LSCS group	LDH group	*P* value
**LF thickness**	5.4 ± 0.6	2.9 ± 0.6	<0.05
**Fibrosis score**	3.1 ± 0.7	2.3 ± 0.8	<0.05
**Leptin**			
mRNA	0.94 ± 0.18	0.85 ± 0.18	<0.05
Protein	0.87 ± 0.32	0.47 ± 0.34	<0.05

Abbreviations: LDH, lumbar disc herniation; LF, ligamentum flavum; LSCS, Lumbar spinal canal stenosis;.

### Effect of leptin on expression of collagen in LF cells

**Figure 5:**
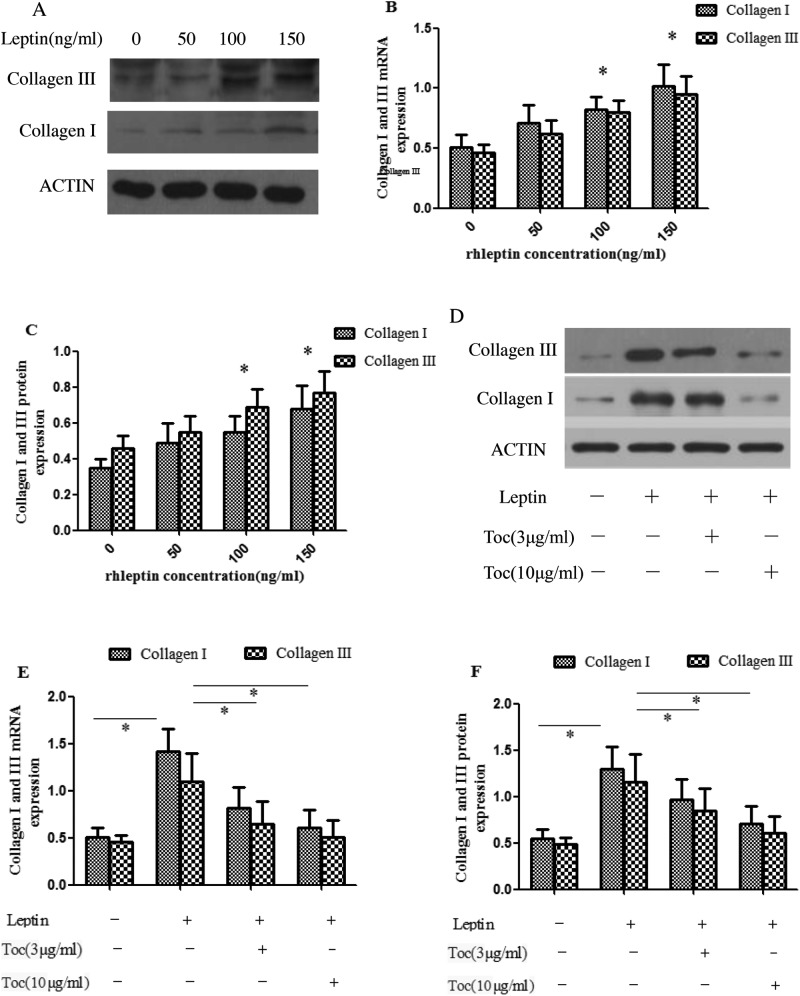
Leptin up-regulated collagen expression in human LF cells by activating IL-6 expression RT-PCR and Western blot analyses showed that the mRNA (**B**) and protein (**A** and **C**) levels of collagen I and collagen III in hrleptin-treated cells were significantly elevated as compared with untreated cells (*P<0.05). Western blot (**D** and **E**) and RT-PCR (**F**) analyses of collagen expression in human LF cells induced by 150 ng/ml hrleptin with or without Tocilizumab (3 and 10 μg/ml). *P*-values were analyzed by one-way ANOVA. All data are representative of three independent experiments and are means ± SEM.

To further confirm that the increase of leptin expression in LF tissue had a causal relationship with LF fibrosis, we analyzed the effect of different concentration of rhleptin on the expression of collagen I and III in LF cells. As shown in Figure 5, the results showed that rhleptin treatment of 24 h increased the mRNA (Figure 5B) and protein (Figure 5A,C) expression of collagen I and III in LF cells (all *P*<0.05). To ascertain the important role of IL-6 in the leptin-induced fibrosis and hypertrophy of LF, we added some inhibitors of IL-6 (Tocilizumab, 3 and 10 μg/ml respectively) *in vitro* experiment and elucidated whether the inhibitor neutralized the effect of leptin on expression of collagens in LF cells. As expected, a pronounced decrease in the leptin-mediated increase in the levels of the collagen I and III mRNA (Figure 5E) and protein (Figure 5D,F) was seen in the presence of Tocilizumab.

### Leptin up-regulated IL-6 expression via the NF-κB p65 pathway

**Figure 6:**
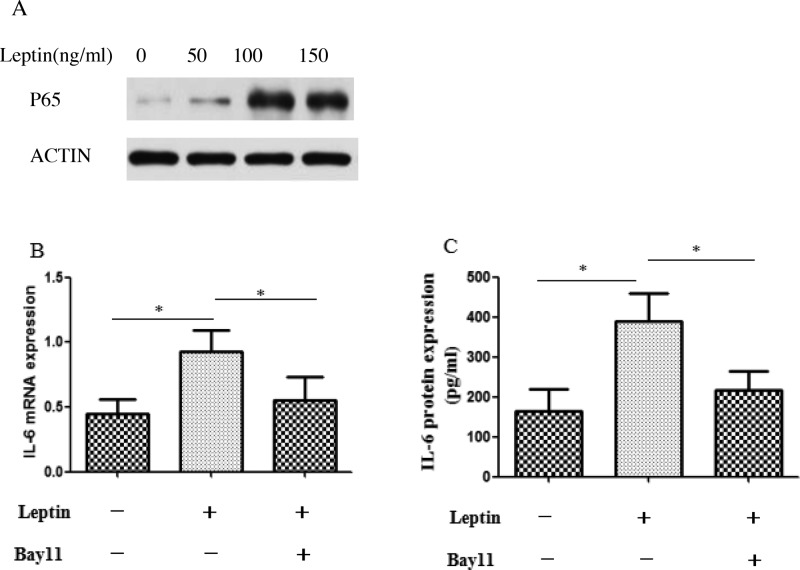
Leptin up-regulated IL-6 expression via the NF-κB pathway (**A**) Western blot analysis of p65 nucleoproteins after treatment of LF cells with hrleptin. (**B**) Quantification of IL-6 mRNA expression in LF cells treated with 100 ng/ml leptin for 24 h with or without Bay 11 (5 μm) for 48 h. (**C**) Quantification of IL-6 secretion in the culture supernatant of human LF cells treated with 100 ng/ml leptin with or without Bay 11 (5 μm). (*P<0.05).

For further investigating the underlying mechanism of leptin-induced LF fibrosis, we analyzed the effect of different concentration of rhleptin on the effect of IL-6 secretion. The results showed that leptin administration elevated IL-6 mRNA (Figure 6A) and protein (Figure 6B) expression (*P*<0.05). To determine whether nuclear factor-κB (NF-κB) signaling is required for leptin induction of IL-6 expression in human LF cells, we first evaluated the activation of NF-κB signaling pathways after treatment with leptin. After treatment with leptin, there was a rapid increase in p65 nucleoprotein levels (Figure 6A). Next, the mRNA expression detected by RT-PCR and the protein expression detected by ELISA all demonstrated that p65 was a crucial adaptor for leptin induced IL-6, as the up-regulated IL-6 expression induced by leptin was significantly attenuated by an NF-κB inhibitor Bay 11 (Figure 6B,C).

## Discussion

Despite the fact that the etiology of LSCS is poorly understood, it is clear that LF hypertrophy is known to be one of the main causes contributing to LSCS [1,2]. The experiment explored in this investigation demonstrated that LF thickness in LSCS group was significantly greater than that in the LDH group. Histologically, the hypertrophied LF lacked any discernible structural arrangement, showing fragmented, disorganized fibers. Moreover, we found LF from LSCS group showed a high fibrosis score. These results showed the fibrotic changes in hypertrophied LF, which were consistent with those reported by previous studies [13,16–19].

Several previous studies regarding LF fibrosis and hypertrophy have implied that many cytokines, including transforming growth factor β1 (TGF-β1) [16], wnt-induced secreted protein-1 (WISP-1) [13], angiopoietin-like protein 2 (Angptl2) [17], lysophosphatidic acid (LPA) [18], and platelet-derived growth factor-BB (PDGF-BB) [19], contribute to the pathologic process of LF fibrosis. Nevertheless, the precise molecular pathogenesis remains largely unknown. As previously stated, fibrosis is a very complicated process. During this process, many cytokines, molecules, and proteins are involved in their regulation [13,16–19]. Therefore, further studies about other factors would help better understand the precise molecular mechanism of LF fibrosis.

It is noteworthy that leptin is a proinflammatory cytokine and plays an important role in the pathogenesis of ARDS, liver, and lung fibrosis [9–11]. For example, leptin-resistant mice are protected from bleomycin-induced pulmonary fibrosis [20]. Also, genetically leptin-deficient ob/ob mice and leptin resistant rats show significantly reduced fibrogenic responses in the liver [21]. Additionally, mice receiving the treatment of recombinant leptin promoted fibrogenic response, whereas leptin receptor-deficient rats failed to develop liver fibrosis [22,23]. Based on these observations, it seemed of interest to examine the effect of leptin on LF fibrosis. Interestingly, we found leptin was abundantly expressed in hypertrophied LF tissue and its expression was significantly correlated with LF thickness and fibrosis score. In addition, for further insight into the function of leptin on LF fibrosis, we isolated the LF cells and observed leptin could up-regulate the expression of collagen I and collagen III in these cells. Previously, it has been well demonstrated that LF fibrosis exhibited progressive increase of collagens I and III in the extracellular matrix [13,16–18]. Given these data, we concluded that leptin might be an important molecule involved in the fibrosis process of LF.

Increasing data suggest that inflammation is one of the important events of the fibrosis phenomenon [24–26]. Blocking the activation of inflammatory responses is proposed as a promising strategy to attenuate cardiac fibrosis [24]. Additionally, it is reported that an increase in the production of proinflammatory cytokines (IL-1, IL-6, and TNF-α) may play a significant role in the processes that leads to excessive scar formation after a burn injury [25]. In agreement with these observations, several studies showed that progressive LF fibrosis and hypertrophy also results from an ongoing chronic inflammatory response [5,26]. Therefore, it can be concluded that inflammatory reaction exhibited an important role in the development of tissue fibrosis.

Leptin can promote an inflammatory response by activating the secretion of proinflammatory cytokines, such as TNF-α, IL-6, and IL-12 in liver fibrosis [27]. The study by Mortensen et al. [28] showed that increased systemic IL-6 was associated with increased inflammation and fibrosis in liver. Recently, it has been shown that IL-6 is significantly increased in LF tissues, which could elevate collagen expression in LF cells, indicating the important role of IL-6 in the pathogenesis of LF fibrosis and hypertrophy [17,26]. In the present study, we isolated the LF cells *in vitro* and observed that leptin could up-regulate the IL-6 expression in these cells, thus suggesting that leptin might be an important molecule causing inflammation in LF tissue by activating IL-6 expression. Moreover, leptin up-regulated IL-6 expression through the NF-κB pathway.

NF-κB is highly activated at sites of inflammation in diverse diseases and can induce transcription of proinflammatory cytokines, chemokines, adhesion molecules, and inducible enzymes, mainly mediating cellular responses to damage, stress, and inflammation [29]. It has been reported that NF-κB plays a crucial role in the production of inflammatory mediators in LF tissues induced by many cytokines or proteins [17,30]. Here, we also found that the NF-κB pathway was essential for leptin-induced IL-6 expression in LF cells.

It is well known that leptin is secreted by adipocytes [20–23]. Therefore, in addition to leptin originated from LF cells demonstrated by our study, we speculated that leptin secreted from the adjacent epidural fat could also influence the fibrosis of LF. Based on the above studies, we proposed the following hypothesis for a potential mechanism in LF fibrosis and hypertrophy. First, an inflammatory reaction would be caused by elevated leptin in LF tissue through activating IL-6 expression. Then, tissue fibrosis would develop due to excessive components of extracellular matrix such as collagen I and collagen III, leading to large structural changes therein. Ultimately, accumulation of the fibrosis would result in LF hypertrophy.

In conclusion, to our knowledge, this is the first paper regarding an association between leptin and LF fibrosis in patients with LSCS. Our findings uncovered a vital detrimental role of leptin in pathogenetic mechanism of LF fibrosis and delineated a potential mechanism of leptin-induced LF fibrosis by activating IL-6 expression, suggesting that down-regulation of leptin might be a promising strategy for treatment of LF fibrosis and hypertrophy.

## Abbreviations

H&E: hematoxylin and eosin
LDH: lumbar disc herniation
LF: ligamentum flavum
LSCS: lumbar spinal canal stenosis
NF-κB: nuclear factor-κB
